# Profiling of Cardiogenic Shock: Incorporating Machine Learning Into Bedside Management

**DOI:** 10.1016/j.jscai.2024.102047

**Published:** 2024-05-28

**Authors:** Elric Zweck, Song Li, Daniel Burkhoff, Navin K. Kapur

**Affiliations:** a The CardioVascular Center, Tufts Medical Center, Boston, Massachusetts; b Department of Cardiology, Pulmonology and Vascular Medicine, Medical Faculty, Heinrich Heine University Duesseldorf, Duesseldorf, Germany; c Medical City Healthcare, Dallas, Texas; d Cardiovascular Research Foundation, New York, New York

**Keywords:** cardiogenic shock, heart failure, machine learning, myocardial infarction, phenotyping

## Abstract

Cardiogenic shock (CS) is a complex clinical syndrome with various etiologies and clinical presentations. Despite advances in therapeutic options, mortality remains high, and clinical trials in the field are complicated in part by the heterogeneity of CS patients. More individualized targeted therapeutic approaches might improve outcomes in CS, but their implementation remains challenging. The present review discusses current and emerging machine learning-based approaches, including unsupervised and supervised learning methods that use real-world clinical data to individualize therapeutic strategies for CS patients. We will discuss the rationale for each approach, potential advantages and disadvantages, and how these strategies can inform clinical trial design and management decisions.

## Introduction

Cardiogenic shock (CS) is a heterogeneous clinical syndrome characterized by impaired cardiac function leading to tissue hypoperfusion^1^ with high short-term mortality rates of 40% to 60%.^2^, ^3^, ^4^, ^5^ Despite intensified research focus on CS in recent years, mortality in CS has not improved significantly.^6^^,^^7^ Major difficulties in the management of CS reside not only in the lack of evidence but also in the lack of tools to rapidly identify and adequately risk stratify patients in CS, which mainly arises from the heterogeneity of CS patients and is rooted in the various etiologies, phenotypes, clinical presentations, and the lack of consistency among parameters and strategies used to accurately assess and monitor shock severity.^8^ The majority of patients experience CS as a cause of either acute myocardial infarction (AMI-CS)^9^ and de novo or acute-on-chronic heart failure (HF-CS).^4^^,^^5^ Recent data indicate that CS patients with AMI-CS or HF-CS exhibit different rates of in-hospital mortality and variable hemodynamic and metabolic responsiveness to therapeutic interventions, which include the use of pulmonary artery catheters, drug therapy, and temporary mechanical circulatory support.^4^^,^^8^^,^^10^ However, a variety of other factors beyond CS etiology are associated with or may even influence the mortality risk, deterioration, effects of drug or device interventions, and recovery in CS patients. These factors include biomarkers (eg, lactate, pH), hemodynamic profiles (eg, right- or left-sided congestion, cardiac output), and demographic characteristics (eg, age, sex) among others.^2^^,^^11^, ^12^, ^13^, ^14^ The plethora of potentially relevant features and their interplay complicates the development of targeted and adequately powered randomized controlled clinical trials in CS. This is illustrated by the fact that even the most contemporary trials have failed to demonstrate any survival benefit with routine use of commonly used temporary mechanical circulatory support devices in CS due to myocardial infarction,^6^^,^^7^^,^^15^ or even any difference with respect to outcomes when comparing different inotropic agents.^16^

Due to these unresolved issues and the apparent need for data-driven improvement in quality of care, machine learning (ML) applications have recently gained particular attention in CS research. ML is a form of artificial intelligence that comprises various types of computational algorithms or statistical models designed to carry out prespecified tasks without being explicitly programmed. These tasks can include pattern recognition, clustering, and dimension reduction (unsupervised ML), as well as prediction (supervised ML) (Central Illustration). A somewhat special case is reinforcement learning which is trained by interacting with an environment, including generative models such as ChatGPT.^17^ With increasing availability of large databases and improved access to advanced ML methods, even more ML applications are expected to come. In this review, we will provide a brief overview of currently proposed ML models for use in clinical CS management and provide an outlook of what is necessary to have ML applications find their way to bedside management and improve outcomes of patients with CS.

**Central Illustration:**
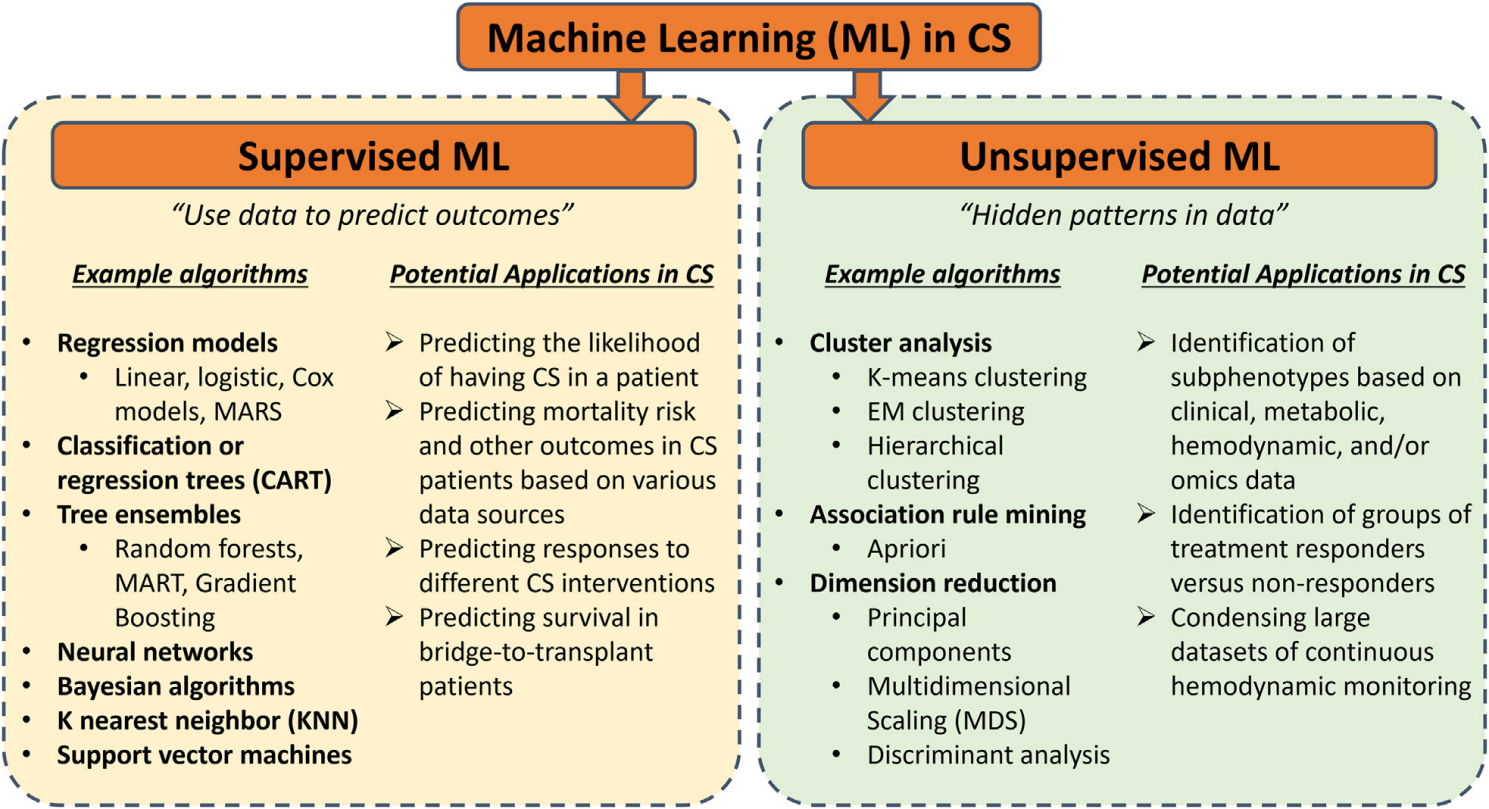
Machine Learning (ML) algorithms with potential applications in cardiogenic shock (CS), divided into supervised and unsupervised ML algorithms.

## Unsupervised ML-assisted phenotyping of CS patients

Clustering algorithms are unsupervised ML algorithms with the aim to distinguish subsets in a dataset with previously unknown categories, by grouping data points that are similar to those within their subset but dissimilar to data points in other subsets. These algorithms have been tested in various diseases which are also hallmarked by heterogeneity and different treatment responses, such as sepsis, acute respiratory distress syndrome, or diabetes mellitus.^18^, ^19^, ^20^, ^21^ The historically most commonly used clustering algorithms in medical research are K-means, hierarchical clustering, and latent class analysis, but a variety of alternative approaches have emerged in recent decades.^22^^,^^23^ Clustering patients with a specific, but heterogeneous disease bears the potential of identifying subsets of patients with more homogeneous clinical characteristics.^23^ In analyses performing such clustering, the resulting subsets have been labeled as subtypes, phenotypes, subphenotypes, or endotypes, and these terms have been used interchangeably in this context.^18^^,^^24^^,^^25^

The incentive to use clustering algorithms based on clinical data of patients is that reproducible phenotypes of disease and their actual use in clinical research and practice could improve medical care by improving the clinicians’ ability to tailor specific interventions to the most responsive subtype of patients, eventually enabling precision medicine.^23^ Although predicting the individual effects of treatment interventions for individual patients would likely be favorable in the sense of personalized medicine, this can hardly be achieved due to the impossibility of conducting randomized controlled trials for each type of population that will be encountered in CS. ML-based phenotypes, however, may account for the heterogeneity of treatment effect seen in randomized controlled trials as their results can be stratified by CS phenotype. For this, phenotypes would potentially allow to inform clinical trial design to test whether a specific phenotype might benefit from 1 intervention more than another. On the other hand, unsupervised ML algorithms cannot be perfectly validated, because the exact number and characteristics of a disease can never be known but only estimated based on the input features and methodology.

**Figure 1:**
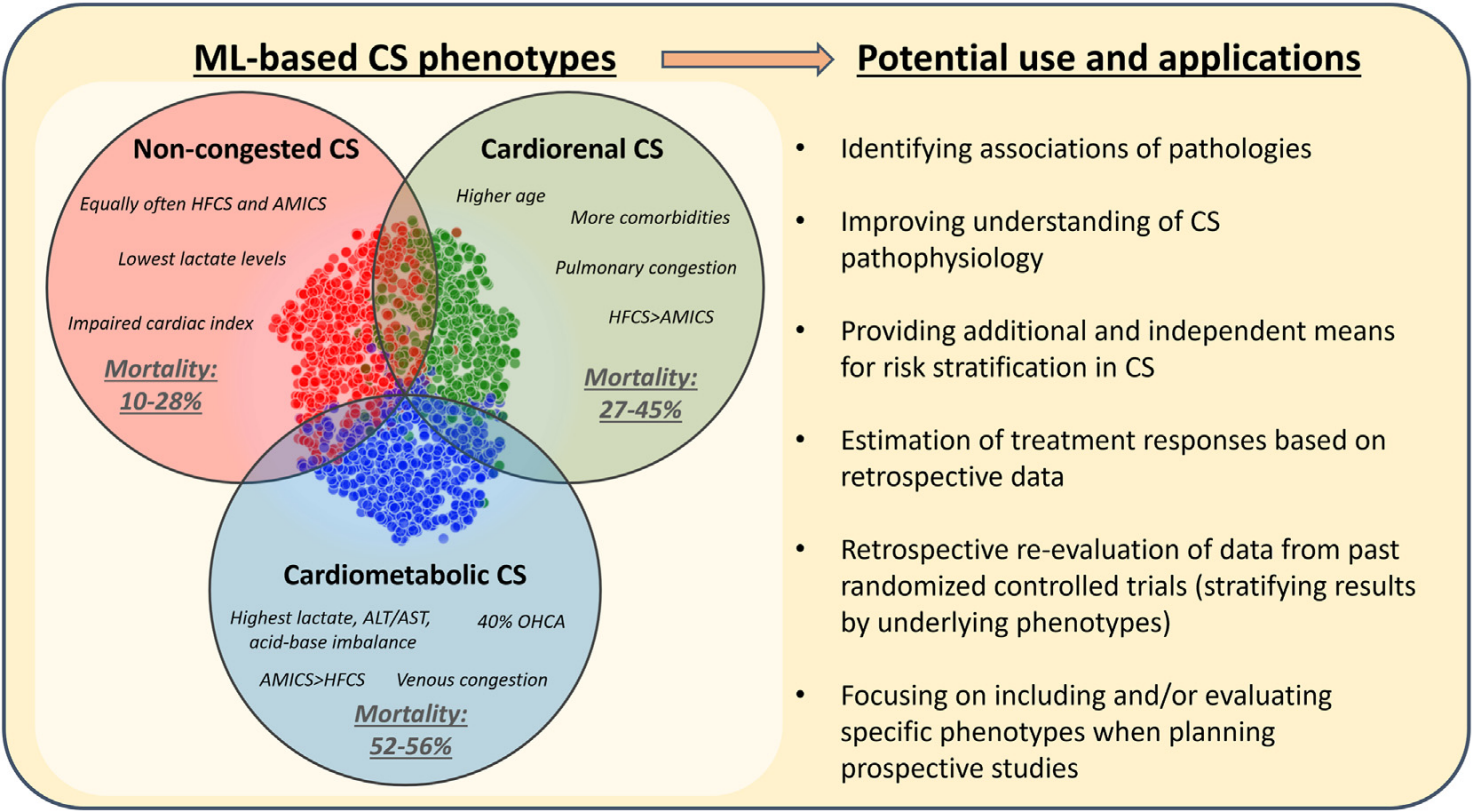
**Selected characteristics and mortality rates of patients within cardiogenic shock (CS) phenotypes as identified in previous reports.**^26^, ^27^, ^28^ AMI, acute myocardial infarction; HF, heart failure; ML, machine learning.

**Figure 2:**
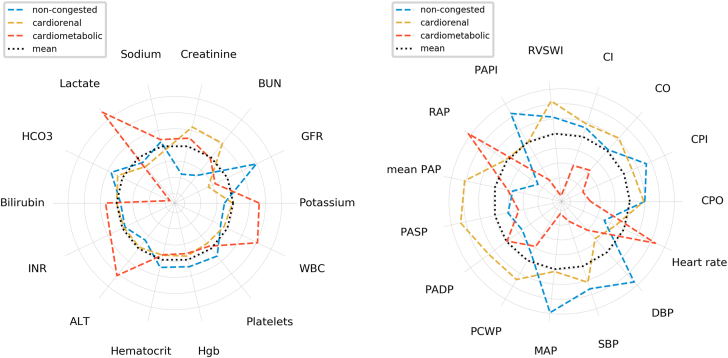
**Figure modified from Zweck et al.**^26^**ALT, alanine aminotransferase; BUN, blood urea nitrogen; CI, cardiac index; CO, cardiac output; CPI, cardiac power index; CPO, cardiac power output; DBP, diastolic blood pressure; GFR, glomerular filtration rate; Hgb, hemoglobin; INR, international normalized ratio; MAP, mean arterial pressure; PADP, pulmonary artery diastolic pressure; PAP, pulmonary artery pressure; PAPI, pulmonary artery pulsatility index; PASP, pulmonary artery systolic pressure; PCWP, pulmonary capillary wedge pressure; RAP, right atrial pressure; RVSWI, right ventricular stroke work index; SBP, systolic blood pressure; SVI, stroke volume index; WBC, white blood cell count**.

A first attempt in clustering patients with CS has been carried out by collaborators from the Cardiogenic Shock Working Group and the Danish Retroshock registries.^26^ Consensus K-means clustering in patients with AMI-CS and HF-CS led to the discovery of 3 reproducible phenotypes with consistently replicable clinical characteristics. Based on the clinical characteristics of patients in these phenotypes, they were labeled “non-congested,” “cardiorenal,” and “cardiometabolic” CS (Figure 1).^26^, ^27^, ^28^ Naturally, these unidimensional labels can only approximate at most the complex interactions of variables that lead to the assignment of a patient to a specific phenotype (Figure 2).^26^ These proposed phenotypes were subsequently tested in other cohorts, as further external validation is required prior to consideration of their implementation in clinical practice (Table 1).^25^, ^26^, ^27^, ^28^, ^29^, ^30^, ^31^, ^32^ Jentzer et al^25^^,^^27^ tested the same and other clustering methods in an external dataset of patients with CS admitted to a US cardiac intensive care unit. In this analysis, 3 phenotypes of CS were also identified with similar characteristics compared to those initially reported, independent of the clustering methodology that was used.^25^ The reproducibility of clinical characteristics and mortality rates of patients in these phenotypes could also be confirmed in a recent external validation analysis of the Cardiogenic Shock Working Group V2 registry.^28^ Another external validation was proposed by Ortega-Hernández et al^33^ who assigned these phenotypes based on simple cutoffs in baseline laboratory variables and assessed their hemodynamic profiles. Wang et al^34^ tried to use consensus K-means clustering in a large cohort of CS patients but could only distinguish 2 phenotypes of CS. The methodology used in that analysis, including missing preprocessing of categorical variables, the high number of variables included for clustering, and the fact that the variables with relevant impact on the clustering mostly included markers of renal function, as well as the lack of an external validation cohort, make these results difficult to interpret. Another group, Yu et al,^31^ performed a latent class analysis on 630 patients from the Medical Information Mart for Intensive Care IV database and also found 3 distinct profiles that showed similar characteristics to those reported by Jentzer et al^27^ and the Cardiogenic Shock Working Group with higher age and renal impairment in the “cardiorenal” phenotype and acid-base balance disturbance in the “cardiometabolic” phenotype.^26^ Recently, a different group (Wang et al)^32^ attempted to retrospectively phenotype a small sample of patients undergoing venoarterial extracorporeal membrane oxygenation therapy and again identified 3 profiles including a profile with metabolic (hepatorenal) impairment and the highest rate of mortality. Finally, Soussi et al^35^ applied latent class analysis retrospectively to a medium-sized prospective multicenter dataset to identify subtypes only among survivors of CS. They found 2 different phenotypes with differences in clinical characteristics as well as 1-year mortality after initial CS hospitalization.

**Table 1:** Approaches to subphenotyping of CS using unsupervised ML.

Data source	CSWG V1 registry: MI	CSWG V1 registry: HF	Danish Retroshock Registry^29^	Mayo Clinic retrospective registry^30^	Mayo Clinic retrospective registry^30^	CSWG V2 registry	MIMIC-IV database	Retrospective single-center cohort in Beijing
CS etiology	AMI	HF	AMI	All-cause	All-cause	All-cause	MI	All-cause, ECMO-supported only
Methodology	Consensus K-means clustering	Consensus K-means clustering and nearest centroid classification	Consensus K-means clustering and nearest centroid classification	K-means clustering	Latent class analysis	Nearest centroid classification	Latent profile analysis	Consensus K-means clustering
Number of patients	410	480	1069	1498	1498	796	630	210
Selected characteristics of phenotype I	“Non-congested,” lowest mortality	“Non-congested,” lowest mortality	“Non-congested,” lowest mortality	“Non-congested,” lowest mortality	“Non-congested,” often had AMI and cardiac arrests, lowest mortality	“Non-congested,” lowest mortality	“baseline” profile, lowest mortality	“platelet preserved,” lowest mortality
Selected characteristics of phenotype II	“cardiorenal,” advanced age, comorbidities, worse kidney function, higher pulmonary artery pressures	“cardiorenal,” advanced age, comorbidities, worse kidney function, higher pulmonary artery pressures	“cardiorenal,” advanced age, comorbidities, worse kidney function	“cardiorenal,” advanced age, comorbidities, chronic diseases, worse kidney function, highest mortality in SCAI stage E	“cardiorenal,” higher proportion of patients non–AMI CS	“cardiorenal,” advanced age, comorbidities, worse kidney function, higher pulmonary artery pressures, higher proportion of patients HF-CS	advanced age, more comorbidities, worse renal function	“hyperinflammatory,” advanced age, comorbidities, worse kidney function
Selected characteristics of phenotype III	“cardiometabolic,” acidosis, high lactate, increased right atrial pressures, highest in-hospital mortality	“cardiometabolic,” acidosis, high lactate, increased right atrial pressures, highest in-hospital mortality	“cardiometabolic,” acidosis, high lactate, highest in-hospital mortality	“hemometabolic,” acidosis, high lactate, lowest stroke volume, highest in-hospital mortality across SCAI stages B-D	“hemometabolic,” often had AMI and cardiac arrests, highest in-hospital mortality	“cardiometabolic,” acidosis, high lactate, increased right atrial pressures, highest in-hospital mortality	Acid-base balance disturbance, systemic inflammatory response, increased right atrial pressures highest in-hospital mortality	“hepatic-renal,” increased transaminases and bilirubin
Source	Zweck et al^26^	Zweck et al^26^	Zweck et al^26^	Jentzer et al^27^	Jentzer et al^25^	Zweck et al^28^	Yu et al^31^	Wang et al^32^

AMI, acute myocardial infarction; CS, cardiogenic shock; CSWG, Cardiogenic Shock Working Group; HF, heart failure; MIMIC, Medical Information Mart for Intensive Care; ML, machine learning; SCAI, Society for Cardiovascular Angiography & Interventions.

Although no unsupervised ML algorithm can provide a definitive answer with respect to how many different subtypes of CS could exist, the above-mentioned articles cumulatively provided robust evidence for reducibility and prognostic impact of the initially reported 3 phenotypes of CS. As noted, the validity of these 3 phenotypes has been confirmed by findings in external cohorts and from the use of different clustering algorithms and input variables in these external cohorts.

Whether 1 of the 3 phenotypes could benefit more from a specific intervention will be a key question for future prospective research (Figure 1). As often demonstrated in previous reports, causal inference is difficult to obtain from nonrandomized data in CS, because it is difficult to retrospectively account for all residual confounding characteristics of patients with CS.^36^ This confounding is likely the reason why in retrospective analyses, use of inotropes, vasopressors, or short-term mechanical circulatory support is associated with increased mortality, whereas this does not translate to respective randomized controlled trials.^2^^,^^37^ A recent retrospective analysis indicates that use of short-term mechanical circulatory support devices is associated with worse outcomes in patients with “cardiorenal” but not in the other phenotypes of CS.^28^ Another report suggests that the “non-congested” phenotype is linked to worse responsiveness to vasopressor use compared to the other phenotypes.^31^ However, more data are needed before specific recommendations can be made regarding which interventions are most effective in specific CS phenotypes.

Another aspect that is apparent in all mentioned analyses of CS clusters is that clustering algorithms have always been applied at only 1 time point, ie, at admission. Thus, it remains unclear, whether CS patients can shift from 1 phenotype to another or whether different CS phenotypes are just a display of different progression of CS. Future studies ought to evaluate this by applying clustering algorithms repetitively throughout hospitalization.

## Supervised ML for early identification and monitoring of CS patients

Early identification of patients with CS, determination of refractory CS, and early intervention in patients not responding to initial treatments are major obstacles in the care of patients with CS. In recent years, team-based approaches have been endorsed by various medical associations to foster improvements in early identification of CS and quicker decision-making.^38^, ^39^, ^40^ Yet, the access to these means varies vastly between centers and their success may be subject to the personal experience of the team members.^41^

Recently, supervised ML tools have been trained to overcome these challenges. In several recent studies, gradient boosting-based or logistic regression models have been used to predict the occurrence of all-cause CS using retrospective data from different US datasets based on ICD codes.^42^, ^43^, ^44^ Another study trained a lasso model to predict late-onset CS after ST-elevation myocardial infarction.^45^ Although the accuracy of the ML models in all of these reports is promising, none of them have provided an adequate external validation cohort; thus, their external validity remains to be shown prior to any considerations of their use in clinical practice. These limitations are in line with ML tools that have been applied to predict all-cause shock or septic shock in intensive care units and have yet to be confirmed in external validation.^46^, ^47^, ^48^, ^49^ Retrospective identification of and distinction of different types of shock comes with inherent limitations that could potentially be overcome in the future with increasing availability of high-quality datasets and prospective retraining of ML algorithms.

To the best of our knowledge, there are no reports of using ML applied to real-time hemodynamic data or imaging data such as echocardiography, waveform analyses, X-rays, or electrocardiograms to predict responses to therapy or the likelihood of further deterioration of the CS state. With the increasing access to digital and online monitoring tools (eg, Harvi,^50^ Impella SmartAssist) with centrally available data, there is the possibility to address such clinically important questions with ML models analyzing large amounts of data.

## ML-based means of predicting outcomes in CS

Estimating the risk of mortality early in the course of CS is critical for timely therapeutic decision-making and for adequate inclusion in randomized controlled trials.^8^^,^^9^ There have been many attempts to risk stratify and predict outcomes of CS patients using standard statistical methods.^51^^,^^52^ The most prominent recent approach for improved risk stratification is the expert consensus-based classification of CS severity proposed by the Society for Cardiovascular Angiography & Interventions (SCAI) and endorsed by other medical associations which has been confirmed to risk stratify CS patients across their hospital stay.^8^^,^^30^^,^^53^ The previously mentioned unsupervised ML-based CS phenotypes have demonstrated their ability to risk stratify beyond and in combination with this SCAI classification system.^26^, ^27^, ^28^

More recently, supervised ML models have been evaluated for this purpose.^54^ Rong et al^55^ and Cha et al^56^ predicted survival in CS using boosted tree models in different datasets but did not provide validation based on applying these models to independent cohorts. A more comprehensive approach was reported by Yamga et al^57^ using large-scale derivation and validation datasets of CS patients with SCAI stage C or greater. Their model employed a simple checklist with decent performance in external validation with a c-statistic of 0.76 (0.73-0.78). Moreover, Stephens et al^58^ used a neural network-based ECMO Predictive Algorithm scoring system whose aim was to predict outcomes of patients under VA-ECMO support independent of the underlying diagnosis, thus including patients without CS. This analysis extended a previous similar report attempting mortality prediction in ECMO patients in a smaller sample of 283 patients.^59^ To which extent these retrospectively trained models will remain valid, particularly in CS patients, after potential shifts in ECMO management due to recent evidence from randomized controlled trials^6^^,^^60^ remains to be evaluated prospectively.

Beyond all-cause CS, there is a considerable number of ML-based models that predict mortality after AMI independent of CS, even though CS may often be on the causal pathway between AMI and death.^61^, ^62^, ^63^, ^64^

The above-mentioned unsupervised ML-based phenotypes also provide additive estimates of in-hospital mortality risk in CS patients. These estimates are less individualized than those provided by supervised ML algorithms but rather provide risk categories with confirmed significant prognostic implications independent of and in combination with the SCAI classification.^26^, ^27^, ^28^ This may be particularly useful when estimating treatment responses, whereas supervised ML algorithms may often require infeasible amounts of data when applied individually.

## Past and future challenges for ML methods in the CS space

### Data sources

**Figure 3:**
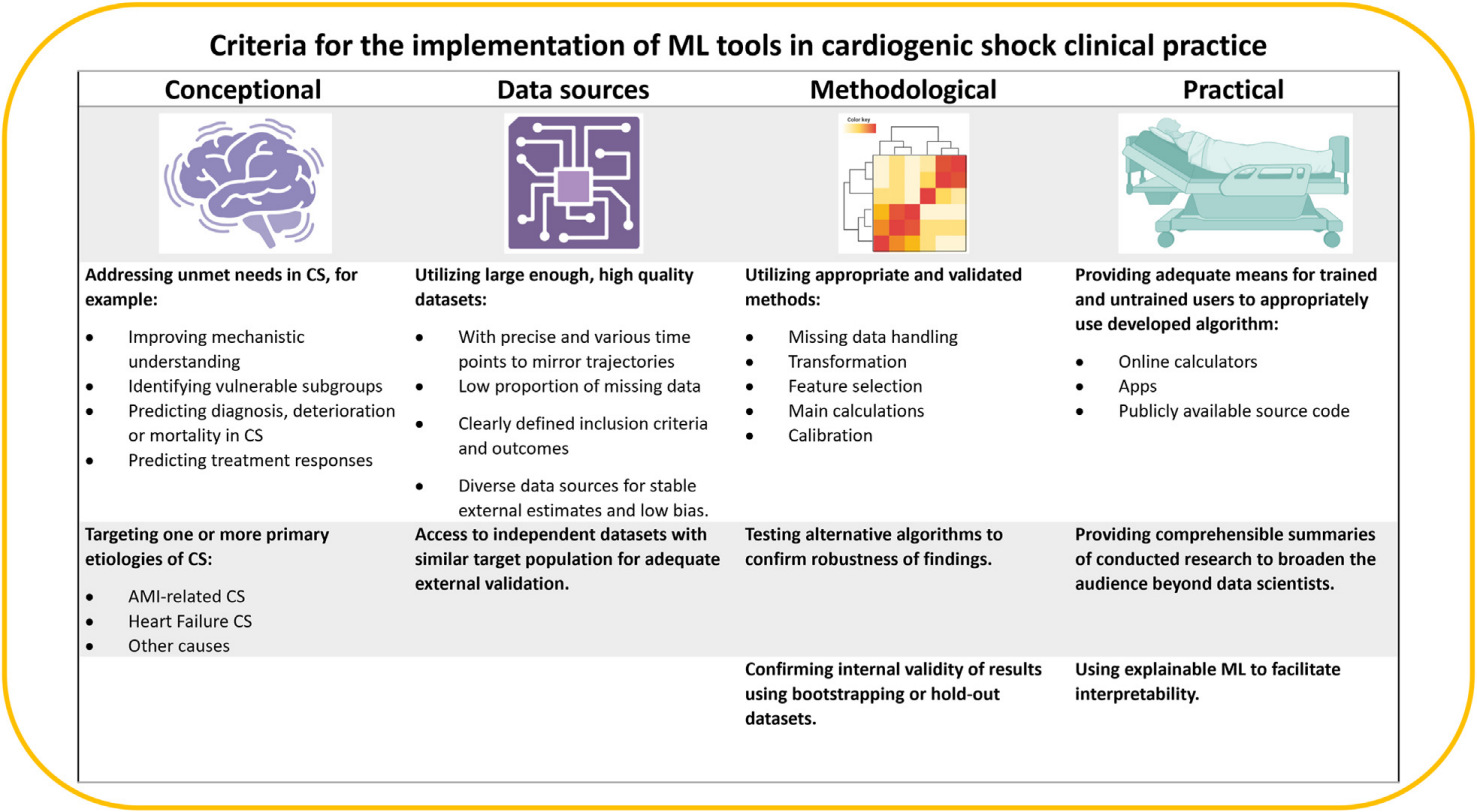
**Criteria for implementation of machine learning (ML) in cardiogenic shock (CS) clinical practice.** AMI, acute myocardial infarction.

Limited availability of representative, adequate, large-scale datasets for the use of ML methods in CS has been an important inhibitor of development in this research area in past decades (Figure 3). Unlike for some other diseases, defining CS retrospectively can be challenging and every definition comes with its own pitfalls. The necessity for retrospective and prospective CS datasets to enable and refine risk stratification has been recognized in recent years, leading to the emergence of several collaborative research efforts to fill this gap.^13^^,^^29^^,^^65^^,^^66^ However, further advances in granularity of datasets and reassessment of data at multiple time points throughout hospitalization will be required for ML methods to be used to their full potential in CS research and practice. In this context, standardized sets of measurements (including laboratory parameters and hemodynamics) across centers are useful to facilitate external applicability of ML algorithms.

### Technical considerations

A major limitation of many studies presented in this review is the inconsistency of the reporting and application of statistical and ML methods. Many of the prediction studies did not provide adequate external validation, lacked sufficient sample size, performed insufficient sensitivity analyses to confirm robustness of the models, or did not provide data on model calibration. These issues with published ML literature have been recognized and previously studied and reviewed systematically.^67^^,^^68^ Comprehensive guidelines on the adequate reporting of ML studies in the medical field are needed and underway, for example, through the TRIPOD+AI initiative.^69^ Yet, it will remain difficult for clinicians and researchers unfamiliar with the methodology to dissect the quality of publications. Even for skilled researchers, it may be difficult to estimate the quality of a model if insufficient data on validation and calibration are provided and the source code cannot be accessed.

Many of the mentioned studies are lacking a demonstration of the validity of the algorithms in suitable external cohorts. Sometimes, data are randomly split into a training and a test cohort, but may not be sufficient to indicate external validity, particularly in monocentric datasets. External validation cohorts need to mirror the same underlying disease as the derivation cohort, which in the case of CS can often be challenging due to varying definitions across countries and centers—at the same time, they should differ enough from the derivation cohort so that replication of the algorithms can confirm more generalizable results. Furthermore, although validation of supervised ML algorithms is usually simply limited to access to the original algorithm, validation of unsupervised ML algorithms is often more challenging, and, in this regard, less consensus exists among experts. Finally, ideal validation does not only occur in retrospective datasets but prospectively, at bedside, which has not been done in any of the above-mentioned reports in the CS space. These limitations highlight the need for collaborative efforts in CS research and reporting guidelines in the space of ML applications in health care.

### Clinical significance

Another issue that applies to both unsupervised and supervised ML reports in the field is the plurality of published papers and models, despite the scarcity of publicly available or generally available large datasets of CS patients with sufficient data quality and granularity. Some of the heretofore-described reports even used the same source data (for example the single-center-based Medical Information Mart for Intensive Care III) with the same question but arrived at different results and conclusions. With that many parallel works, it is imperative to put the added value of newly proposed AI models into question. Is it just an alternative statistical approach yielding merely the same results as prior approaches? Or does it really have the potential to advance risk stratification or understanding of CS? More collaborative initiatives on CS are required globally to address these issues and avoid parallel computing and publication.

### Practical considerations: The transition from “bench” to bedside

From a clinical perspective, the noted plurality of publications is particularly worrying. How is a clinician going to decide which of the various prediction models to apply? For many clinicians, the answer could be to not use any ML model at all because they cannot distinguish those models based on high-qualitative methods from others. This hesitation is understandable as even CS guidelines have so far avoided recommendations for the use of ML methods in clinical practice. For other clinicians, the answer to which ML model to use will likely be largely dependent on the accessibility of the models through calculators or checklists. But simpler models tend to come with reduced discriminative ability and the tradeoff between bias and variance can come into play.

Finally, with these obstacles in mind, it remains speculative whether clinical decision-making based on ML model output is superior to conventional human decision-making based on randomized trials and clinical experience in CS. This could be addressed in prospective implementation studies or questionnaires for physicians or patients.

To have ML models implemented into clinical practice, they will need to be useful in real-time and be available at the bedside^70^—preferably without the necessity for a human user to manually enter data into a calculator. In this context, an integration of ML algorithms in electronic health records will be necessary for larger-scale use of ML tools; however, this integration may come with its own limitations. As such, automatic importing of numerical data may not be accurate (for example hemodynamic data with short-term fluctuations), informative missing values (for example ML-based interpretation of measurements that have not been obtained based on retrospective information), and issues with parsing unstructured data. The implementation of adequate interfaces to facilitate timely and efficient ML-based decision-making will be 1 major obstacle in upcoming years particularly in health care systems with no or only partial use of electronic health records.

The main goals to improve usability and benefit from ML applications in CS include the following: (1) development of integrative ML algorithms that can synthesize and analyze multimodal data as present in health care, including tabular data, physical sensor data (hemodynamic monitoring), images, sound, and videos; (2) interconnection of machines, devices, sensors, and users to facilitate real-time processing of medical data and thus medical decision-making; (3) wide-spread source data and code accessibility allowing for harmonization of various scoring systems and data sources; (4) the ability of cyber-physical systems to perform micromanagement tasks through decentralized decision-making reducing the need for human intervention. The main challenges, if overcome, could dramatically transform the quality of ML applications in CS, are the barriers to data use, exchange and the repeated publishing of stand-alone ML algorithms instead of collaboration on a larger scale to synthesize data inputs and algorithms which could eventually lead to the generation of close-to-ideal models. Approaches partially addressing these issues include decentralized learning and swarm learning.^71^

## Conclusion

Both, unsupervised and supervised ML techniques come with the general ability to perform calculations related to the major questions in the care of patients with CS: when is a patient in CS? What is the most likely prognostic phenotype? Will the patient recover under current therapy or deteriorate? Will the patient survive? Yet, the plurality and varying quality of reported ML models as well as difficulties in access and bedside implementation represent barriers to realizing the clinical benefit emerging from these models. Still, in the long run, ML-assisted improved phenotyping and prognostication of CS patients could assist clinical decision-making and eventually help improve outcomes in CS.
